# Old lessons learned anew: family-based methods for detecting genes responsible for quantitative and qualitative traits in the Genetic Analysis Workshop 17 mini-exome sequence data

**DOI:** 10.1186/1753-6561-5-S9-S83

**Published:** 2011-11-29

**Authors:** Claire L Simpson, Cristina M Justice, Mera Krishnan, Robert Wojciechowski, Heejong Sung, Jerry Cai, Tiffany Green, Deyana Lewis, Dana Behneman, Alexander F Wilson, Joan E Bailey-Wilson

**Affiliations:** 1 Statistical Genetics Section, Inherited Disease Research Branch, National Human Genome Research Institute, National Institutes of Health, 31 Center Drive, 333 Cassell Drive Suite 1200, Baltimore, MD 21224, USA; 2 Genometrics Section, Inherited Disease Research Branch, National Human Genome Research Institute, National Institutes of Health, 31 Center Drive, 333 Cassell Drive Suite 1200, Baltimore, MD 21224, USA

## Abstract

Family-based study designs are again becoming popular as new next-generation sequencing technologies make whole-exome and whole-genome sequencing projects economically and temporally feasible. Here we evaluate the statistical properties of linkage analyses and family-based tests of association for the Genetic Analysis Workshop 17 mini-exome sequence data. Based on our results, the linkage methods using relative pairs or nuclear families had low power, with the best results coming from variance components linkage analysis in nuclear families and Elston-Stewart model-based linkage analysis in extended pedigrees. For family-based tests of association, both ASSOC and ROMP performed well for genes with large effects, but ROMP had the advantage of not requiring parental genotypes in the analysis. For the linkage analyses we conclude that genome-wide significance levels appear to control type I error well but that “suggestive” significance levels do not. Methods that make use of the extended pedigrees are well powered to detect major loci segregating in the families even when there is substantial genetic heterogeneity and the trait is mainly polygenic. However, large numbers of such pedigrees will be necessary to detect all major loci. The family-based tests of association found the same major loci as the linkage analyses and detected low-frequency loci with moderate effect sizes, but control of type I error was not as stringent.

## Background

Family studies have been an integral part of genetic research since the 1950s. This useful and powerful study design fell out of favor with the advent of genome-wide association studies (GWAS), which focus on unrelated, population-based study designs. However, advances in the field of next-generation sequencing techniques have resurrected family-based designs as an alternative to the population-based approach. In this study, we evaluate methods from the linkage analysis and family-based association analysis era and apply these methods to the analysis of the Genetic Analysis Workshop 17 (GAW17) mini-exome sequence data [1]. For the family-based tests of association, we use one of the new approaches for analyzing rare variation in sequence data, using several variations of the collapsing method of Li and Leal [2]. After analyses were planned and under way, we requested the generating models for the simulated trait data so that we could determine whether our analyses detected any of the “true” causal sequence variants (SVs) for each simulated trait.

## Methods

In the simulation process, the 202 founders of the eight extended pedigrees were a random sample from the 697 individuals in the simulated unrelated data set, so only a subset of the total trait-generating SVs were present in the family data, with rare variants being particularly underrepresented [1]. The simulated SV genotypes were identical for all 200 replicates. Phenotypes were simulated using the same age, sex, and causal SVs in all replicates; smoking status differed across replicates. Thus power and type I error rates may be affected by the dependence of these values across replicates. All SVs were tested for Hardy-Weinberg equilibrium. SVs that were monomorphic or had missing heterozygotes were removed. SVs not in Hardy-Weinberg equilibrium were flagged for further consideration. Allele frequencies were estimated with Sib-Pair [3] using maximum-likelihood estimation and were used in all linkage analyses of the SV data. For linkage analysis, the traits were adjusted for covariates that had a significant effect on the trait (Age and Smoking for Q1; none for Q2; Age, Sex, and Smoking for Q4). Haseman-Elston regression, Lander-Green nonparametric linkage analysis, and Elston-Stewart parametric linkage analysis were performed on replicate 1 only, whereas variance components (VC) analyses were performed with all 200 replicates. For the association analyses, the three quantitative traits in each replicate were adjusted for Age, Sex, and Smoking and were centered using linear regression, with the residuals being used in all association analyses.

### Linkage analysis in relative pairs using provided identity-by-descent sharing data

Pairwise identity-by-descent (IBD) sharing values were provided by GAW17 for a fully informative marker at the location of each gene in the mini-exome data. Because the actual genotypes of this set of highly informative linkage markers were not provided, we were not able to analyze the fully informative marker data using the standard linkage programs used for the SVs. Instead, we used a modified Haseman-Elston regression to analyze relative pairs. We selected sib pairs and grandparent-grandchild pairs based on phenotype data from replicate 1. For the qualitative trait, we selected all the discordant sib pairs and a subset of concordant affected and concordant unaffected sib pairs. For the quantitative traits we selected all possible sib pairs. We analyzed all possible grandparent-grandchild pairs. In replicate 1, we performed standard logistic regression of the qualitative trait (concordantly affected pair = 0, concordantly unaffected pair = 0, discordant pair = 1) and standard linear regression of the squared difference in phenotype values of the members of each pair for the quantitative traits, using the provided IBD sharing values for the pairs as the independent variables.

### Linkage analysis of SVs in nuclear families

Because of the large size of the pedigrees, the Lander-Green algorithm implemented in Merlin could not analyze the entire pedigrees. Pedigrees were broken down into nuclear families with Sib-Pair [3]. We analyzed all the nonmonomorphic SVs in the entire data set and also split the set of subpedigrees into one file for each pedigree, to get overall nonparametric linkage and LOD scores for each of the eight families. We performed standard nonparametric single-point linkage analysis in Merlin on all traits in replicate 1. In addition, we used whole-genome multipoint VC linkage analyses of Q1 and Q2 phenotypes using Merlin-VC and the SV genotypes over all 200 replicates. The power to detect phenotype-generating markers using VC was estimated empirically as the proportion of replicates that showed suggestive (LOD ≥ 2) or genome-wide significant (GWS) (LOD ≥ 3.4) evidence of linkage at the marker locus. Type-I error rates for Q1 and Q2 were estimated as the proportion of nongenerating markers in which the VC linkage LOD scores exceeded these thresholds using SVs only on chromosomes that did not contain causal variants.

### Linkage analyses of causal SVs in extended pedigrees

The Elston-Stewart algorithm in FastLink [4,5] is able to handle larger pedigrees than the Lander-Green algorithm. We analyzed all causal nonmonomorphic SVs in replicate 1. We performed parametric single-point linkage analysis on the qualitative trait assuming a model with a disease allele frequency of 0.01 and penetrances of 0.5 and 0.05 for genotypes *DD*/*Dd* and *dd*, respectively. For Q1, the model was frequency of *D* = 0.01, mean *DD* = 1, mean *Dd* = 0, mean *dd* = −1, common variance = 1. The number of causal loci that exhibited GWS evidence of linkage (LOD ≥ 3.3) and suggestive linkage (LOD ≥ 1.9) are reported. Time and computer program constraints did not allow for multipoint analysis on replicate 1 or for single-point analysis to be performed on all replicates.

### Rare variant coding for association analyses

Genotypes for 13,784 nonmonomorphic sequence variants were used as given (uncollapsed), coded as the number of minor alleles, and also with rare variants collapsed by a method based on the work of Li and Leal [2] and Morris and Zeggini [6] (see Dering et al. [7] for a review of these methods). Rare variants for collapsing were defined using four different criteria: (1) minor allele frequency (MAF) < 1%; (2) MAF < 1% and nonsynonymous SV; (3) MAF < 5%; and (4) MAF < 5% and nonsynonymous SV. Genomic regions for collapsing were defined by location within a gene. Collapsed rare variants were coded by the presence or absence of any rare SV within the given genomic region. Variants not classified as rare remained coded as the number of minor alleles. The MAF from each variant was calculated for the pooled data across ethnic groups.

### Tests of association in parent-offspring trios

We used ROMPrev version0.2 [8], to estimate trait and locus-specific heritabilities and to test association between each quantitative trait and each SV in all 200 replicates using all possible parent-child trios. In this method, genotyping of the parents is not required, substantially reducing sequencing costs. Data from the same four rare variant collapsing definitions were analyzed. Genome-wide significant *p*-values were obtained by performing a Bonferroni correction based on the number of SV markers analyzed per collapsing method (uncollapsed: *p* < 3.6 × 10^−6^; MAF < 1%: *p* < 5.1 × 10^−6^; MAF < 1% and nonsynonymous SV: *p* < 4.24 × 10^−6^; MAF < 5%: *p* < 7.54 × 10^−6^; MAF < 5% and nonsynonymous SV: *p* < 4.85 × 10^−6^).

### Tests of association in extended pedigrees

We used ASSOC [9] in the extended pedigrees to test for association between the adjusted quantitative traits Q1, Q2, and Q4 and the SV genotypes, using the uncollapsed genotypes and all four collapsing strategies. We performed a test for additive genotypic effect and examined the results from the likelihood-ratio test. SVs were considered GWS in the same way as in the ROMP analyses.

## Results

The family data did not include all possible causal and noncausal variants because of selection resulting from random sampling of the founders [1]. In the family data, there were 13,780 monomorphic SVs and 13,784 nonmonomorphic SVs (17 of 39 nonmonomorphic causal SVs for Q1, 29 of 72 causal SVs for Q2, and 12 of 51 causal SVs for the qualitative trait). SVs in *VEGFA* and *VEGFC* were the only nonmonomorphic causal variants with a major effect (generating model *β* > 1) on Q1. *BCHE* contained the only nonmonomorphic (but rare) SV with a major effect on Q2. There were no nonmonomorphic SVs with major effects on the qualitative trait except for the three SVs acting through the effects of Q1 and Q2 on the qualitative trait.

### Linkage analysis in relative pairs using provided IBD sharing data

For replicate 1 for Q1 and Q2 using sib pairs and grandparent-grandchild pairs, we observed many peaks greater than the 2.2 threshold for suggestive linkage for sib pairs [10] or the 1.9 threshold for grandparent-grandchild pairs [10], but all were type I errors. We did not observe any GWS linkage (Table 1).

**Table 1:** Linkage analyses using classic methods in replicate 1

	Haseman-Elston regression			
			
Gene	Sib pairs	Grandparent-grandchild pairs	Lander-Green (Merlin)	Elston-Stewart (FastLink)
Qualitative trait				
False positives	0 (0)	0 (2)	0 (0)	NA
Quantitative trait Q1				
*VEGFA*	0 (0)	0 (0)	0 (1)	1 (0)
*VEGFC*	0 (0)	0 (0)	0 (0)	1 (0)
False positives	0 (0)	0 (6)	0 (3)	NA
Quantitative trait Q2				
False positives	0 (0)	0 (0)	0 (0)	NA
Quantitative trait Q4				
False positives	0 (7)	0 (2)	0 (1)	NA

Number of signals detected at genome-wide significant levels, with suggestive levels in parentheses.

### Linkage analysis of SVs in nuclear families

For the Lander-Green single-point linkage analysis in Merlin using SVs, for the nonparametric analysis of nuclear families in replicate 1 for Q1, three SVs had *p*-values less than the 1 × 10^−3^ threshold for suggestive linkage, but only one signal was due to a causal variant. For Q2, no SVs were significant at *p* = 1 × 10^−3^. In Q4, one SV was significant at *p* < 1 × 10^−3^. Again, no GWS results were observed (Table 1).

For the VC linkage analysis in Merlin-VC using SVs, *VEGFA* and *VEGFC* were GWS in 25% and 6.5% of replicates, respectively, using nuclear families (Table 2). Overall, 92.5% and 27.5% of replicates showed at least one false-positive suggestive linkage signal at noncausal markers for Q1 and Q2, respectively. However, only 12% (Q1) and 0.5% (Q2) of replicates showed at least one false-positive GWS linkage signal.

For the linkage analysis of causal SVs in extended pedigrees, only two SVs (C6S2981 in *VEGFA* and C4S4935 in *VEGFC*) had a major locus effect (*β* > 1) on Q1 in these families. In replicate 1, using single-point Elson-Stewart linkage analyses in complete pedigrees, both SVs were detected at genome-wide significance (LOD > 3.3; for *VEGFA* with the original Q1 trait and for *VEGFC* with the residual of Q1 after adjusting for Age, Sex, and Smoking by means of linear regression). However, for both rare and common variants with smaller effect sizes on Q1, no suggestive or GWS linkage signals were detectable in replicate 1 in these eight large families. No SVs that contributed to the qualitative trait were detected at genome-wide significance (the most positive LOD was 1.45 for the causal variant in *VEGFC*, which affected the qualitative trait through the effect of Q1 on disease risk) (Table 1).

### Tests of association in parent-offspring trios

The results of family-based association analyses using ROMP are shown in Table 2. Given the similarity of the results obtained for all collapsing definitions used, we show the results only for collapsing definition 2 (MAF < 1% and nonsynonymous SV). For Q1, the number of SVs that were GWS per replicate ranged from 7 to 117 for collapsing definition 2, with true signals corresponding to *FLT1*, *KDR*, *VEGFA*, and *VEGFC* (Table 2). *VEGFA* and *VEGFC* were GWS in all replicates. For Q2, the number of GWS SVs per replicate ranged from 0 to 13. GWS SVs in at least two replicates corresponded to causal variants in *LPL*, *SREBF1*, *VLDLR*, *VNN1*, and *SIRT1*. The mean false-positive rate across all replicates was 0.3761% for Q1, 0.0065% for Q2, and 0.0021% for Q4.

**Table 2:** Linkage and association analyses in all 200 replicates

Gene	Causal SV	MAF	*β*^a^	Merlin-VC	ROMP	ASSOC
Q1						
*FLT1*	c13s431	0.0172	0.7414		1 (0.5%)	32 (16%)
*FLT1*	c13s523	0.0667	0.6500		7 (3.5%)	75 (37.5%)
*FLT1*	CSV^b^					1 (0.5%)
*KDR*	c4s1878	0.1650	0.1357		18 (9%)	10 (5%)
*KDR*	c4s1884	0.0208	0.2956		2 (1%)	
*VEGFC*	c4s4935	0.0007	1.3573	13 (6.5%)	200 (100%)	200 (100%)
*VEGFA*	c6s2981	0.0022	1.2065	50 (25%)	200 (100%)	200 (100%)
Q2						
*LPL*	c8s442	0.01578	0.49459		11 (5.5%)	15 (7.5%)
*SIRT1*	c10s3109	0.00072	0.51421		4 (2%)	
*SREBF1*	c17s1043	0.0043	0.49941		2 (1%)	4 (2%)
*VLDLR*	CSV^b^				4 (2%)	1 (0.5%)
*VNN1*	c6s5380	0.17073	0.24437		4 (2%)	10 (5%)
*VNN3*	c6s5441	0.09828	0.27053		1 (0.5%)	3 (1.5%)

Number (%) of replicates are shown where the test achieved genome-wide significance. ASSOC and ROMP results are presented for collapsing definition 2 (MAF < 1% and nonsynonymous SV).

^a^ Effect size of SV on quantitative trait [1].

^b^ Multiple causal variants exist in this gene’s collapsed sequence variant.

### Tests of association in extended pedigrees

The results of family-based association analyses using ASSOC are shown in Table 2. Given the similarity of the results obtained for all collapsing definitions used, we show the results only for collapsing definition 2 (MAF < 1% and nonsynonymous SV). For Q1, the number of SVs that were GWS per replicate ranged from 2 to 25, for which the causal variants detected corresponded to *FLT1*, *KDR*, *VEGFC*, and *VEGFA* (Table 2). *VEGFC* and *VEGFA* were GWS in all 200 replicates. For Q2, the number of GWS SVs per replicate ranged from 0 to 2. SVs significant in at least two replicates were all causal variants in *LPL*, *SREBF1*, *VNN3*, and *VNN1*. The mean false-positive rate across all replicates was 0.0358% for Q1, 0.0006% for Q2, and 0.0007% for Q4.

## Discussion

The sample size of this simulated data set was not large enough to have high power for linkage analysis in sib pairs, grandparent-grandchild pairs, or nuclear families using standard parametric and nonparametric analysis. Variance components analysis using nuclear families had some ability to detect the SVs with the largest effect sizes in Q1 at suggestive levels but not at GWS levels. Parametric two-point linkage in complete pedigrees provided evidence of GWS linkage (in replicate 1) for the two major loci for Q1 that had variants segregating in the families. Although time constraints did not allow this analysis to be performed on all replicates, comparison of this result to the total lack of power in sib pairs, grandparent-grandchild pairs, and nuclear families in replicate 1 suggests that the well-known improvement in linkage power when using extended pedigrees holds true for analysis of SVs with major gene effects, even in the presence of extreme genetic heterogeneity and polygenic effects. In all linkage methods except VC analysis, false-positive rates were well controlled at GWS levels but not at suggestive levels, another classic result. More large pedigrees would be needed to find the major loci not represented in these families as a result of locus heterogeneity and to detect moderate effect loci, but these results show that linkage can be powerful to detect oligogenic effects on variance of a quantitative trait even when the causal variant is rare in the population.

Family-based tests of association were powerful only for detecting association of genes that contained SVs of large effect in this small number of families. However, they did detect some SVs of moderate effect in a small percentage of replicates. Larger sample sizes should improve power. An important observation is that one GWS type I error was found in 50% of replicates, suggesting that associations that are only occasionally replicated may still be false positives. ROMP and ASSOC had comparable results. ROMP has the advantage that it does not require genotyping of the parents, which would greatly reduce sequencing costs. However, ROMP had more GWS false positives because it treats related trios as independent. The type I error rate in ROMP is a known problem that can be addressed easily with permutation testing because of the speed of the method.

## Conclusions

One of the approaches suggested for the analysis of high-throughput sequencing data in families is to use extended pedigrees and classic linkage analysis methods for rare variant sequence analysis. This appears to be useful for detecting major loci and for controlling type I error. However, certain aspects of these methods are problematic. First, for most current multipoint linkage methods, intermarker linkage disequilibrium must be removed by dropping single-nucleotide polymorphisms, if all the founders in the family are not genotyped. Second, the combination of a large number of markers with large pedigree sizes is cumbersome. Two-point linkage using the Elston-Stewart algorithm performed well, but most implementations do not easily allow analysis of large numbers of markers and this algorithm is not computationally feasible for more than two or three markers in multipoint linkage. Large extended pedigrees are most powerful for detecting major loci, but the Lander-Green algorithm, which is able to handle large numbers of markers in multipoint linkage, does not scale well for large pedigree sizes. In real data, the large size of pedigrees with a full exome- or genome-wide set of variants would represent a significant analytical challenge. Variance components methods on extended pedigrees using SOLAR or methods that calculate approximations to Elston-Stewart likelihoods, such as SimWalk2, are computationally challenging but may be feasible for sequence data in extended pedigrees given powerful computing resources. More method development is clearly needed.

Overall, the ASSOC and ROMP association methods appear promising. Family-based association tests, which can leverage both family structure and linkage disequilibrium to their advantage, were able to find the same loci as the linkage analyses. Again, those variants with the largest effect sizes were the ones detected at GWS levels. Reducing the significance threshold inflated the false-positive rate without increasing the number of real signals found. Our results underline the importance of correcting for multiple testing and replicating significant results for control of family-wise error.

Family-based association study designs offer several advantages over population-based designs: better control of population stratification, enrichment of rare variants, and the ability to discriminate relevant variants that segregate with the trait. The downside to these designs is that many analysis methods such as ASSOC, although powerful, require sequencing of many family members, which increases the cost of analysis. Some family-based association methods can reduce sequencing costs by using only parent-offspring trios or distantly related affected pairs. For continuous traits, ROMP is a powerful method that reduces the number of individuals who need to be sequenced, because it requires only phenotype information on the parents.

For traits such as those simulated for GAW17, which exhibit extreme genetic heterogeneity and mostly polygenic effects on variation, a much larger sample would be required to detect more of the causal SVs using either linkage or family-based association methods. The GAW17 simulation assumed that exome sequencing was performed on 697 individuals in 8 extended pedigrees. At current whole-exome sequencing prices, this would be an expensive study. However, sequencing costs are falling rapidly. It is not unreasonable to believe that within the next few years the costs of a whole-exome sequence will fall below $1,000 and may approach $500 per person, making large family studies feasible. Sequencing a few distant relatives, followed by genotyping of all shared rare variants, is an alternative strategy that can take advantage of the strengths of family-based sequencing studies while controlling costs.

## Competing interests

The authors declare that there are no competing interests.

## Authors’ contributions

All authors contributed to the design of the study. CLS did the HWE tests and estimated allele frequencies in the families, performed the Haseman-Elston regression (assisted by TG and DL), split the pedigrees into nuclear pedigrees, performed Lander-Green linkage analyses. RW performed the variance-components linkage analysis. JEBW performed the Elston-Stewart linkage analyses. CMJ carried out the ASSOC family based association analyses. MK performed the ROMP family based association analyses. AFW and CMJ planned the family based association analyses. HS collapsed the rare variants for the association analyses. JC produced the residuals of the traits adjusted for the covariates. CLS and CMJ drafted the paper, all authors edited the drafts and read and approved the final manuscript.
